# Tumor Necrosis Factor Alpha Induces Reactivation of Human Cytomegalovirus Independently of Myeloid Cell Differentiation following Posttranscriptional Establishment of Latency

**DOI:** 10.1128/mBio.01560-18

**Published:** 2018-09-11

**Authors:** Eleonora Forte, Suchitra Swaminathan, Mark W. Schroeder, Jeong Yeon Kim, Scott S. Terhune, Mary Hummel

**Affiliations:** a Department of Surgery, Comprehensive Transplant Center, Northwestern University Feinberg School of Medicine, Chicago, Illinois, USA; b Department of Medicine, Division of Rheumatology, Northwestern University Feinberg School of Medicine, Chicago, Illinois, USA; c Department of Microbiology and Immunology, Medical College of Wisconsin, Milwaukee, Wisconsin, USA; d Biotechnology and Bioengineering Center, Medical College of Wisconsin, Milwaukee, Wisconsin, USA; Princeton University

**Keywords:** cytomegalovirus, latency, reactivation

## Abstract

We used the Kasumi-3 model to study human cytomegalovirus (HCMV) latency and reactivation in myeloid progenitor cells. Kasumi-3 cells were infected with HCMV strain TB40/E*wt*-GFP, flow sorted for green fluorescent protein-positive (GFP^+^) cells, and cultured for various times to monitor establishment of latency, as judged by repression of viral gene expression (RNA/DNA ratio) and loss of virus production. We found that, in the vast majority of cells, latency was established posttranscriptionally in the GFP^+^ infected cells: transcription was initially turned on and then turned off. We also found that some of the GFP^−^ cells were infected, suggesting that latency might be established in these cells at the outset of infection. We were not able to test this hypothesis because some GFP^−^ cells expressed lytic genes and thus it was not possible to separate them from GFP^−^ quiescent cells. In addition, we found that the pattern of expression of lytic genes that have been associated with latency, including UL138, US28, and RNA2.7, was the same as that of other lytic genes, indicating that there was no preferential expression of these genes once latency was established. We confirmed previous studies showing that tumor necrosis factor alpha (TNF-α) induced reactivation of infectious virus, and by analyzing expression of the progenitor cell marker CD34 as well as myeloid cell differentiation markers in IE^+^ cells after treatment with TNF-α, we showed that TNF-α induced transcriptional reactivation of IE gene expression independently of differentiation. TNF-α-mediated reactivation in Kasumi-3 cells was correlated with activation of NF-κB, KAP-1, and ATM.

## Abstract (precis)

HCMV is an important human pathogen that establishes lifelong latent infection in myeloid progenitor cells and reactivates frequently to cause significant disease in immunocompromised people. Our observation that viral gene expression is first turned on and then turned off to establish latency suggests that there is a host defense, which may be myeloid cell specific, responsible for transcriptional silencing of viral gene expression. Our observation that TNF-α induces reactivation independently of differentiation provides insight into molecular mechanisms that control reactivation.

## INTRODUCTION

Human cytomegalovirus (HCMV) is a ubiquitous human pathogen of the betaherpesvirus family. Infection is transmitted through contact with body fluids, including saliva, urine, blood, and genital secretions. Hematogenous spread of the virus leads to a systemic infection, with multiple cell types infected in many organs. In immunocompetent hosts, primary infection is typically subclinical and is resolved by activation of innate and adaptive immunity. However, latently infected cells, which carry viral DNA but do not produce infectious virus, are able to escape immune surveillance and can persist for the life of the host.

Under the appropriate conditions, the virus in these cells wakes up from its dormant state to reactivate the infectious cycle. In immunocompetent hosts, viral replication is controlled by the immune response, but reactivation of latent virus can be a significant infectious complication in immunocompromised hosts, such as recipients of solid organ or bone marrow transplants. Reactivation of CMV in solid organ transplant recipients is associated with increased risk of CMV disease, acute and chronic allograft rejection, infection with other opportunistic pathogens, graft failure, and death (1). Risk factors for reactivation include CMV serostatus of the donor (D) and recipient (R), with the highest risk in the D^+^/R^−^ combination; specific immunosuppression protocols; and inflammatory conditions associated with high tumor necrosis factor alpha (TNF-α) secretion, including allograft rejection and sepsis (1).

The cell type harboring latent CMV DNA in solid organs has not been clearly identified. However, it is known that latent virus is transmitted through blood transfusion, and monocytes in the blood and hematopoietic progenitor cells (HPCs) in the bone marrow are sites of latency *in vivo* (2–10). Experimental models have shown that these cells are less permissive to lytic replication and that they support a latent infection (11–16). Cell-type-specific establishment of latency is thought to be due to a combination of host and viral factors. Infection activates a host intrinsic immune response, which recognizes viral DNA invading the nucleus and silences viral gene expression at the outset of infection through heterochromatinization of viral genomes (13, 17–26). Factors present in the viral particle, including the tegument protein pp71, enter the cell upon infection and counteract this host defense response to activate viral gene expression. In cells that support latency, pp71 is sequestered in the cytoplasm and is therefore unable to perform this function (26–28). Differentiation of myeloid cells to dendritic cells increases permissiveness of these cells to infection and also induces reactivation of both naturally and experimentally latently infected cells (12, 13, 15, 20, 29–32). Thus, the generally accepted paradigm is that myeloid progenitor cells are not permissive to infection due to heterochromatinization and transcriptional repression of invading viral genomes. The process of myeloid cell differentiation is thought to change the balance of repressive and activating cellular factors that control both viral and cellular transcription, resulting in changes to the viral epigenome that activate viral gene expression (17, 33, 34).

However, some recent observations suggest that the current paradigm may be in need of revision. First, several investigators have demonstrated that, although myeloid progenitors may be less permissive than other cell types, some expression of viral lytic genes is detectable at early times postinfection in various models of latency, including primary CD34^+^ HPCs; Kasumi-3 cells, a CD34^+^ myeloblastic cell line derived from a patient with acute myeloid leukemia (AML) (35), which is a tractable model for CMV latency and reactivation (27, 36, 37); and primary CD14^+^ peripheral blood mononuclear cells (11, 12, 36–43). Expression of these genes is lost as latency is established. These observations suggest that latency is not necessarily established at the outset of infection but may also be established following activation of viral transcription.

Second, differentiation of myeloid cells into dendritic cells *in vivo* occurs in the context of infection and inflammation (44), and all of the physiologically relevant differentiation factors that have been used to induce reactivation of CMV in primary hematopoietic cells, including granulocyte-macrophage colony-stimulating factor (GM-CSF) (45), TNF-α, interleukin-6 (IL-6), and lipopolysaccharide (LPS), are mediators of inflammation. Thus, it is difficult to distinguish the roles of inflammation versus differentiation in these models. In contrast, Kasumi-3 cells, like other transformed cell lines, are refractory to normal physiological differentiation cues and therefore can be used to dissect the contributions of these two pathways. Here, we have used the Kasumi-3 model to further investigate these issues.

## RESULTS

### Latency is established after activation of transcription.

**FIG 1:**
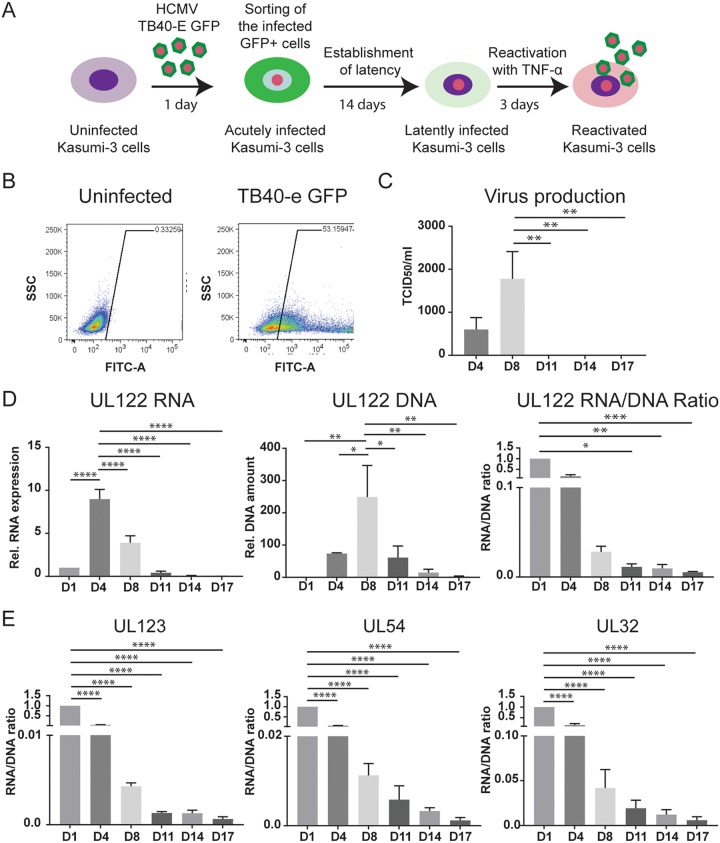
HCMV latency is established after activation of transcription at 14 days postinfection. (A) Schematic outlining the infection model used for studies of latency and reactivation in Kasumi-3 cells. GFP^+^ infected cells were purified by flow cytometry at 1 dpi. On day 14, latently infected cells were treated with TNF-α for 3 days to induce reactivation. (B) Representative FACS analysis of GFP expression in Kasumi-3 infected cells at 1 dpi compared to uninfected cells. FITC, fluorescein isothiocyanate; SSC, side scatter. (C) Release of viral particles into the medium was measured by a TCID_50_ assay on MRC-5 cells after 2 weeks. (D) UL122 mRNA expression and DNA amount were analyzed at the indicated times postinfection and expressed relative to day 1 after normalization to GAPDH or RNase P. (E) RNA/DNA ratios of UL123, UL54, and UL32 over the course of infection. For panels C to E, statistical significance was calculated by a one-way analysis of variance with Dunnett’s multiple-comparison test (*n* = 4). The error bars represent standard errors of the means, and the asterisks indicate *P* values (*, *P* ≤ 0.05; **, *P* ≤ 0.01; ***, *P* ≤ 0.001; ****, *P* ≤ 0.0001) calculated by the comparison to the peak (day 4 for RNA, day 8 for DNA and virus, and day 1 for the RNA/DNA ratio).

The time at which latency is established following infection with HCMV has not been clearly determined. Some previous studies have shown that primary CD34^+^ myeloid progenitor cells do not express lytic viral genes upon infection but rather that viral genomes in these cells are inactivated upon entry and remain quiescent until they receive signals that induce them to differentiate (26, 46). However, other studies have shown that both primary CD34^+^ cells and Kasumi-3 cells express a simian virus 40 (SV40) promoter-driven green fluorescent protein (GFP) reporter, as well as viral genes at early times postinfection (11, 36–41), and that latency can be established in the GFP^+^ population following cell sorting (11, 36, 38, 40, 41, 47). To investigate the question of timing further, we infected Kasumi-3 cells with the TB40/E*wt*-GFP strain of HCMV (48) and analyzed the cells by fluorescence-activated cell sorting (FACS) after 24 h (Fig. 1A). GFP expression was observed in 20 to 50% of cells (Fig. 1B), as previously reported (36). Following inactivation of input virus, we purified the GFP^+^ population by cell sorting and analyzed viral RNA expression, DNA copy number, and production of infectious virus over the course of infection. Latency has been operationally defined as the lack of virus production in cells that carry viral DNA with the capacity to reactivate (49). Production of infectious virus was detectable at 4 days postinfection (dpi) and peaked at 8 dpi but was no longer detectable at 11 dpi (Fig. 1C).

A second characteristic of latency is transcriptional repression of genes involved in lytic replication. We therefore analyzed expression of viral genes representative of the immediate early (IE2, UL122, and IE1, UL123), early (viral DNA polymerase UL54), and late (UL32) phases of the viral life cycle. Expression of these genes peaked at 4 dpi and then fell throughout the course of infection. Viral DNA copy number also increased following infection, peaked 4 to 8 dpi, and then decreased significantly (Fig. 1D; see also Fig. S1 in the supplemental material). Because both viral DNA and RNA copy number dropped over the course of infection, analysis of RNA expression alone does not indicate repression of viral transcription. We therefore calculated the ratio of viral RNA to DNA. Relative to 1 dpi, this ratio fell for all genes from 4 to 14 dpi (Fig. 1D and E). TNF-α induced reactivation of infectious virus in these cells (see below). It is important to note that, because the cells were sorted for GFP expression to obtain a pure population of infected cells in which viral genomes were transcriptionally active, these results show that latency can be established by shutting off gene expression after it has been turned on. We have introduced the term posttranscriptional latency to describe this state.

10.1128/mBio.01560-18.2 FIG S1  Analysis of viral RNA and DNA over the course of infection in Kasumi-3 cells. RNAs and DNAs from representative immediate early, early, and late genes were analyzed by reverse transcription-qPCR and qPCR, respectively, at various times postinfection as described in Fig. 1 and Materials and Methods. Download FIG S1, PDF file, 0.9 MB. Copyright © 2018 Forte et al. 2018 Forte et al. This content is distributed under the terms of the Creative Commons Attribution 4.0 International license.

### Some GFP^−^ cells contain viral DNA and express lytic transcripts.

**FIG 2:**
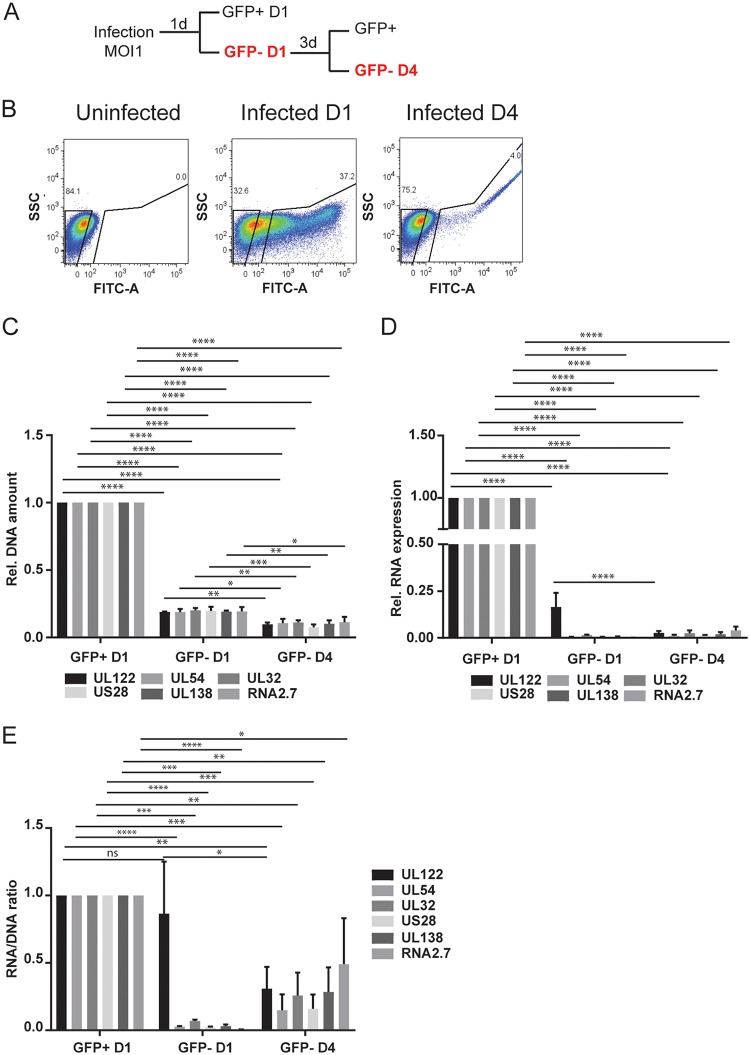
Analysis of GFP^−^ infected cells at 1 and 4 dpi. (A) Schematic outlining the sorting strategy to purify GFP^−^ cells at day 1 and day 4 postinfection. (B) Representative FACS analysis of GFP sorting at day 1 and day 4 postinfection. (C to E) DNA amount (C), mRNA expression (D), and RNA/DNA ratio (E) of UL122, UL54, UL32, US28, UL138, and RNA2.7 analyzed at the indicated times postinfection and expressed relative to day 1. RNA expression values were normalized to GAPDH, and the DNA amount was normalized to RNase P. The statistical significance was calculated by two-way analysis of variance with Tukey’s multiple-comparison test (*n* = 3). The error bars represent the standard errors of the means, and the asterisks indicate *P* values (*, *P* ≤ 0.05; **, *P* ≤ 0.01; ***, *P* ≤ 0.001; ****, *P* ≤ 0.0001) calculated by comparison of the different genes at each time point.

Although our studies indicate that latency can be established posttranscriptionally, we wondered whether there might be a second population of cells in which latency is established at the outset of infection. These cells would carry viral DNA but would not express GFP or viral genes. To test this hypothesis, we analyzed both the GFP-positive and GFP-negative populations for the presence of viral DNA at 1 dpi. To preclude cross-contamination of these fractions, the gates for sorting were set far apart. Quantitative PCR (qPCR) analysis revealed that, although the vast majority of the DNA was present in the GFP^+^ cells, some DNA was detectable in the GFP^−^ population (Fig. 2C). We then analyzed the RNA from these cells for expression of immediate early, early, and late genes (Fig. 2D). Expression of immediate early (IE2, UL122) RNA was detectable in the GFP^−^ population, albeit at a lower level than in the GFP^+^ cells. When we calculated the ratio of UL122 RNA to DNA, we found that there was no difference between the GFP^+^ and GFP^−^ cells, indicating that transcription of the IE genes was equally active in these two populations at this time (Fig. 2E). However, when we analyzed the RNA/DNA ratios of early (UL54) and late (UL32) genes, we found that this ratio was significantly lower in the GFP^−^ cells (Fig. 2E), indicating that these classes of genes were transcriptionally repressed in the GFP^−^ cells. We then analyzed the GFP^−^ cells for changes in GFP expression, viral RNA expression, and DNA copy number over time. We found that some GFP^−^ cells become GFP^+^ by 4 dpi (Fig. 2B). Expression of all classes of viral RNA and DNA copy number increased in these cells from 1 to 4 dpi and then declined after 7 dpi (Fig. S2).

10.1128/mBio.01560-18.3 FIG S2  Kinetic analysis of HCMV DNA and RNA expression in GFP^−^ cells isolated at 1 dpi. (A to C) Relative mRNA expression and DNA amount of UL122, UL54, and UL32 analyzed by reverse transcription-qPCR and qPCR, respectively, at the indicated times postinfection and expressed relative to day 1. Statistical significance was calculated by a one-way analysis of variance with Dunnett’s multiple-comparison test (*n* = 3). The error bars represent the standard errors of the means, and the asterisks indicate *P* values (*, *P* ≤ 0.05; **, *P* ≤ 0.01; ***, *P* ≤ 0.001; ****, *P* ≤ 0.0001) calculated by comparison to the peak at day 4. Download FIG S2, PDF file, 0.8 MB. Copyright © 2018 Forte et al. 2018 Forte et al. This content is distributed under the terms of the Creative Commons Attribution 4.0 International license.

In order to determine whether there was a subset of cells in the GFP^−^ population containing quiescent viral DNA, we resorted these cells at 4 dpi into GFP-positive and -negative cells for analysis of viral DNA and RNA (Fig. 2A and B). The results show that viral DNA was detectable in the GFP^−^ population at 4 dpi (Fig. 2C, GFP-D4). Analysis of RNA expression showed that some expression of lytic genes was still detectable in the GFP^−^ population at 4 dpi (Fig. 2D, GFP-D4). We conclude that (i) the GFP reporter is regulated as an early gene in TB40/E*wt*-GFP-infected Kasumi-3 cells, as previously noted in fibroblasts (50); (ii) there is heterogeneity in the kinetics of viral gene expression after infection of Kasumi-3 cells, with activation of early and late gene expression delayed in some cells; and (iii) it is not possible to use sorting for GFP expression to determine whether a small minority of Kasumi-3 cells infected with TB40/E*wt*-GFP contain viral genomes but remain quiescent.

### US28, UL138, and 2.7-kb RNAs are not differentially expressed in latently infected Kasumi-3 cells.

**FIG 3:**
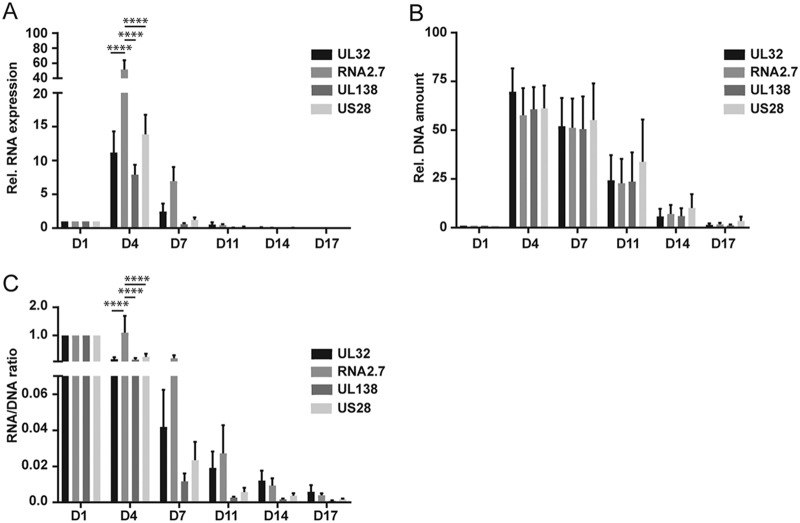
Expression of latency-associated RNAs. mRNA expression (A), DNA amount (B), and RNA/DNA ratio (C) of UL32, RNA2.7, UL138, and US28 analyzed at the indicated times postinfection and expressed relative to day 1. RNA expression values were normalized to GAPDH, and the DNA amount was normalized to RNase P. The statistical significance was calculated by two-way analysis of variance with Tukey’s multiple-comparison test (*n* = 3). The error bars represent the standard errors of the means, and the asterisks indicate *P* values (*, *P* ≤ 0.05; **, *P* ≤ 0.01; ***, *P* ≤ 0.001; ****, *P* ≤ 0.0001) calculated by comparison of the different genes at each time point.

A number of studies have analyzed the viral transcriptome in latency, and some of these have indicated that some lytic genes are also specifically expressed in latency, including US28, UL138, and a noncoding 2.7-kb RNA (RNA2.7) (12, 14, 37, 46, 47, 49, 51–53). We analyzed expression of these genes in GFP^+^ cells over the course of infection in Kasumi-3 cells. RNA2.7 is expressed at very high levels during lytic infection of fibroblasts (54) and at early times after infection of Kasumi-3 cells (Fig. 3A). UL138 and US28 RNAs are also relatively abundant transcripts during lytic infection. To analyze repression of these genes relative to a lytic gene, we analyzed UL32, a gene that is expressed at levels similar to UL138 and US28 at 4 dpi. Our results show that expression of UL138, US28, and RNA2.7 followed the same pattern as that observed for UL32, with a peak of expression at 4 dpi, followed by a significant decrease at 7 dpi (Fig. 3A). When we analyzed the RNA/DNA ratio, we found that expression of these genes was repressed over the course of infection, with kinetics similar to that of UL32 (Fig. 3C). The RNA/DNA ratio of RNA2.7 was significantly higher than other genes at 4 dpi, consistent with its high level of expression during lytic infection, but there was no difference between UL32 and UL138 or US28 at any time point, and there were no differences in the RNA/DNA ratios between UL32 and any of the other genes analyzed after 4 dpi (Fig. 3C). Thus, there was no preferential expression of UL138, US28, or RNA2.7 over the lytic gene UL32 when latency was established.

### TNF-α induces reactivation of HCMV in Kasumi-3 cells independently of myeloid cell differentiation.

**FIG 4:**
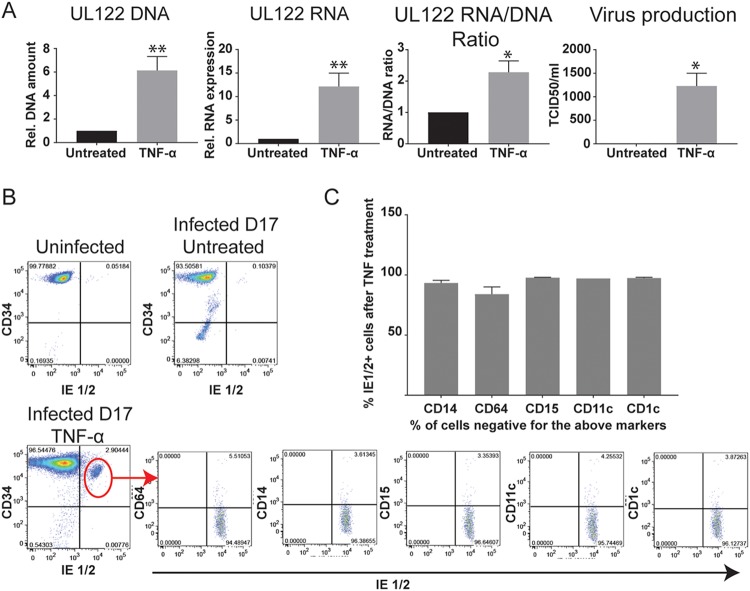
TNF-α induces reactivation independently of differentiation. (A) Latently infected cells (14 dpi) were treated or not with TNF-α for 3 days. On day 17, DNA and RNA from infected cells were analyzed for DNA copy number and expression of viral genes as decribed in Materials and Methods. The release of viral particles in the supernatant was measured by a TCID_50_ assay. The statistical significance was calculated by unpaired *t* test (*n* = 4). The error bars represent the standard errors of the means, and the asterisks indicate *P* values (*, *P* < 0.05; **, *P* < 0.01). (B) Representative FACS analysis of uninfected cells, latently infected cells, and TNF-α-treated cells for the expression of the hematopoietic progenitor marker CD34 and the viral immediate early proteins IE1/2. Reactivating cells were immunophenotyped by gating on the CD34^+^ and IE1/2^+^ population and analyzing expression of markers of myeloid differentiation (CD64, CD14, CD15, CD11c, and CD1c). (C) Percentage of undifferentiated cells within the IE1/2-expressing population in the TNF-α-treated cells (CD14^−^, CD64^−^, CD15^−^, CD11c^−^, and CD1c^−^). The error bars represent the standard errors of the means calculated from 3 experiments.

Previous studies showed that TNF-α induced reactivation of HCMV in latently infected Kasumi-3 cells (36). We confirmed this result using slightly different conditions. Kasumi-3 cells were infected with TB40/E*wt*-GFP virus at a multiplicity of infection (MOI) of 1 to 2, flow sorted for GFP^+^ cells, and cultured for 14 days to establish latency. The cells were then divided into different pools and incubated for a further 3 days in the presence of 5 ng/ml TNF-α or left untreated. Relative to untreated controls, TNF-α induced statistically significant increases in viral DNA copy, RNA expression, and the RNA/DNA ratio, as well as production of infectious virus (Fig. 4A and additional RNA analyses of early and late genes in Fig. S3). In addition, we analyzed the percentage of reactivating cells by flow cytometry after staining with an IE1/2-specific antibody. Expression of IE proteins was essentially undetectable in latently infected, untreated Kasumi-3 cells (Fig. 4B, upper right panel). Treatment with TNF-α reproducibly induced expression of IE1/2 proteins in 2 to 3% of the cells (representative results shown in Fig. 4B, lower left panel).

10.1128/mBio.01560-18.4 FIG S3  TNF induces expression of HCMV early and late genes. RNAs from the experiments shown in Fig. 4 were analyzed for relative expression of the early gene UL54 and the late gene UL32. Download FIG S3, PDF file, 0.8 MB. Copyright © 2018 Forte et al. 2018 Forte et al. This content is distributed under the terms of the Creative Commons Attribution 4.0 International license.

Differentiation of myeloid progenitor cells to a dendritic cell phenotype has long been associated with transcriptional activation of HCMV IE gene expression and reactivation of infectious virus (13, 20, 34, 40). Transformed cell lines are generally resistant to differentiation, and therefore, it seemed unlikely that differentiation would play a role in this model. However, the fact that reactivation occurred in only a small percentage of latently infected Kasumi-3 cells left open the possibility that reactivation occurred in a small number of cells with the capacity to differentiate. To investigate this question, we used immunophenotyping to characterize the differentiation state of Kasumi-3 cells carrying reactivating virus. Latently infected TNF-α-treated or untreated cells were stained for IE1/2 antibody in combination with the hematopoietic progenitor marker CD34 and for markers of various stages of myeloid differentiation, including CD64, a marker of granulomonocytic lineage commitment (55), CD14 (monocytes), CD15 (granulocytes), and CD1c and CD11c, which identify major subpopulations of human myeloid dendritic cells (56). Uninfected Kasumi-3 cells are homogeneous with respect to CD34 expression and do not express other markers of myeloid cell differentiation (Fig. 4B, upper left, and S^4^). Expression of CD34 was lost in a small percentage of cells after establishment of latency, but latent cells did not differentiate (Fig. S4B). All the latently infected cells were negative for expression of IE proteins (Fig. 4B, upper right panel). Two percent to 3% of the cells became IE^+^ after treatment with TNF-α, and these cells retained expression of CD34 (Fig. 4B, lower left panel). We then gated on the IE^+^/CD34^+^ population to analyze expression of differentiation markers in reactivating cells. Approximately 95% of the IE^+^/CD34^+^ cells were negative for CD64 and for markers of mature monocytes and dendritic cells (Fig. 4C). Thus, our data show that differentiation was not required for transcriptional reactivation of IE gene expression in this model.

10.1128/mBio.01560-18.5 FIG S4  Phenotyping of uninfected and latently infected Kasumi-3 cells. Representative FACS analysis of uninfected (A) and latently infected (B) cells for the expression of the hematopoietic progenitor marker CD34 and markers of myeloid differentiation (CD64, CD14, CD15, CD11c, and CD1c). Download FIG S4, PDF file, 1 MB. Copyright © 2018 Forte et al. 2018 Forte et al. This content is distributed under the terms of the Creative Commons Attribution 4.0 International license.

### TNF-α induces activation of NF-κB and a DNA damage response in Kasumi-3 cells.

**FIG 5:**
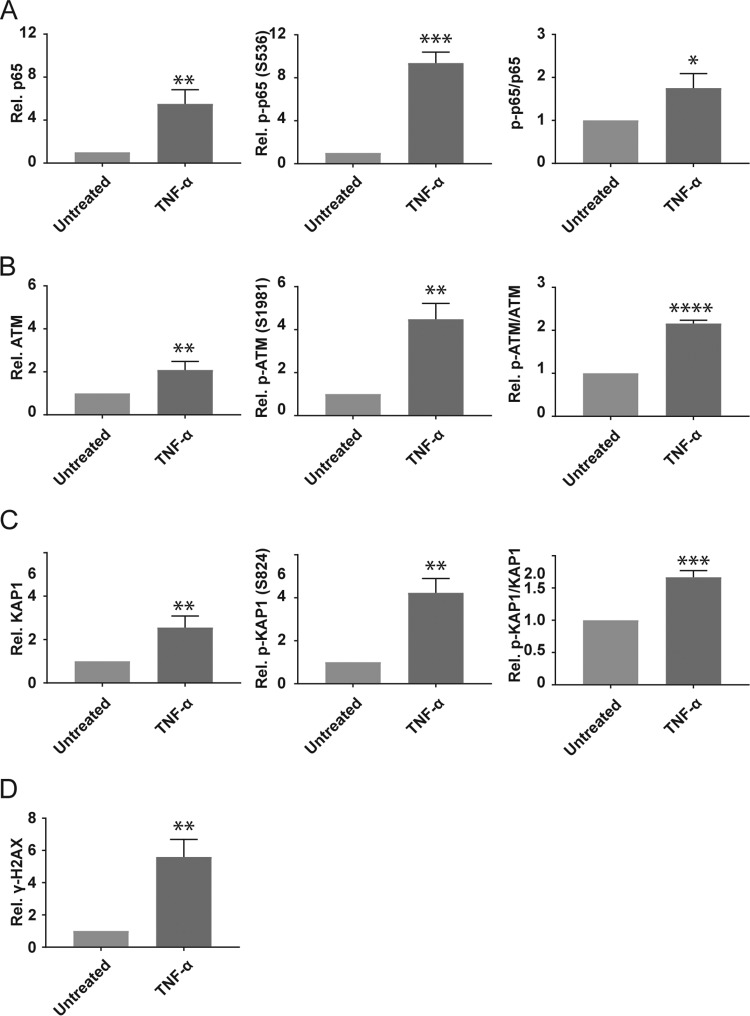
TNF-α-mediated reactivation is correlated with activation of NF-κB and ATM signaling. Latently infected cells (14 dpi) were treated for 3 days with or without TNF-α at 5 ng/ml. (A to D) Flow cytometry analysis was performed to assess the activation of NF-κB (p65) and DDR (ATM, KAP-1, and γ-H2AX). The activation of p65, ATM, and KAP-1 was expressed as the ratio of phosphoprotein to total protein in comparison with untreated cells (p-p65-S536/p65, pATM-S1981/ATM, and p-Kap-S824/Kap1). γ-H2AX is expressed as the ratio of mean fluorescence intensity compared to untreated cells. The error bars represent the standard errors of the means calculated from 3 independent experiments (*, *P* ≤ 0.05; **, *P* ≤ 0.01; ***, *P* ≤ 0.001).

Binding of TNF-α to the TNFR1 receptor activates signaling cascades, which lead to activation of transcription factors that control activation of the HCMV major immediate early promoter (MIEP), including canonical NF-κB (p65/p50) (57–59). Because the IE genes are transcriptionally silent in latently infected cells and they are required for activation of lytic replication, activation of the MIEP is thought to be a requisite first step in reactivation of the virus. Activation of NF-κB is controlled at multiple levels, including cellular localization and posttranslational modification at multiple sites (60). TNF/TNFR1-mediated activation of IκB kinase (IKK) leads to degradation of the inhibitory IκB subunit, which permits translocation of the active p65/p50 complex from the cytoplasm into the nucleus. In addition, IKK mediates phosphorylation of p65 S536 (61, 62), which leads to enhanced transactivation through increased CBP/p300 binding and acetylation of p65 K310 (63). We therefore analyzed total levels of p65 and p-p65 S536 and the ratio of p-p65 S536 to total p65 in both uninfected and latently infected Kasumi-3 cells treated with or without TNF-α (Fig. 5A). Our results show that all of these increased following treatment with TNF-α in latently infected cells.

Previous studies showed that TNF-α induces activation of ATM (64) and that activation of ATM is sufficient to induce reactivation of HCMV in primary hematopoietic progenitor cells through phosphorylation of KAP-1 (S824) bound to latent viral genomes (40). We therefore analyzed total and phospho-ATM (S1981) and total and phospho-KAP-1 (S824) and the ratio of phosphorylated to total proteins. In addition, we analyzed phosphorylation of H2AX (γ-H2AX), a variant histone that is recruited to sites of DNA damage (65). Our results show that all of these markers of DNA damage are increased by treatment of Kasumi-3 cells with TNF-α (Fig. 5B to D).

## DISCUSSION

### Mechanisms of establishment of latency.

HCMV establishes latency in myeloid progenitor cells through heterochromatinization and repression of viral gene expression, but the timing and molecular mechanisms that control this process are not well understood. While some studies have shown that viral gene expression is silenced immediately after infection (26, 46), others have shown that viral gene expression is activated in various models at early times postinfection and that latency is subsequently established in this population of cells (11, 36, 38, 40, 41, 47). Our studies are in agreement with the latter observation. We found that ~90% of infected Kasumi-3 cells become GFP^+^ at 1 dpi. From 4 to 8 dpi, expression of all classes of viral genes was activated in this population, viral DNA was amplified, and infectious virus was produced. At later times, viral RNA expression, DNA copy number, and the RNA/DNA ratio decreased. Some lytically infected cells die after infection, and the drop in RNA/DNA ratio may be due in part to loss of these cells. However, we observed a significant decrease in RNA/DNA ratio between live GFP^+^ cells isolated at 1 dpi, prior to robust viral replication, and those isolated at 17 dpi, when the cells maintain viral DNA but expression of viral genes is nearly undetectable. This difference is due at least in part to transcriptional repression of viral genes during the transition from a lytic to latent infection.

Thus, we find that, although activation of the MIEP is lower in myeloid progenitor cells than permissive fibroblasts (26), Kasumi-3 cells support the full cycle of lytic replication. In contrast to fibroblasts, where the infection is propagated throughout the culture to kill all the cells, our studies suggest that some infected Kasumi-3 cells are able to shut down viral replication and survive. Our studies are in agreement with previous studies of Kasumi-3 cells, which showed increased expression of lytic RNAs and amplification of viral DNA relative to early times postinfection and, in some cases, production of infectious virus, followed by transcriptional repression (36, 37, 66). Although Kasumi-3 cells may be more permissive than primary CD34^+^ HPCs, previous studies have also shown activation of viral gene expression in HPCs prior to establishment of latency (11, 38, 41, 47).

Recent studies indicate that posttranscriptional establishment of latency may be a more general feature of herpesvirus latency (67–70). Previous studies have shown that activation of the immediate early ICP0 promoter was detectable in some but not all neurons of mice latently infected with herpes simplex virus (HSV) (68). Activation of the late gC promoter was also detectable in the acute stage of infection but not in latently infected mice. This observation suggests that, although some viral genes may be expressed prior to establishment of latency, there is a point of no return, and full replication of the virus is not compatible with survival. Interestingly, these authors also demonstrated activation of the HCMV MIEP prior to establishment of latency in neurons infected with HSV.

We considered the possibility that there may be two routes to establishment of latency: repression prior to activation of gene expression and posttranscriptional repression. Our results show that ~10% of the infected cells are GFP^−^ at 1 dpi. We found that there is heterogeneity in the kinetics of viral gene expression and that some GFP^−^ cells become GFP^+^, activate the early and late phases of viral gene expression, and amplify viral DNA with delayed kinetics. Previous studies showed that initiation of HCMV gene expression requires that the cells be in the G_0_ or G_1_ phase of the cell cycle (71), and heterogeneity in cell cycle phase may account for some of the observed variation in the kinetics of gene expression. The majority of the cells that were GFP^−^ at 1 dpi remain GFP^−^ at 4 dpi. But, because some GFP^−^ cells expressed lytic viral transcripts, we were unable to determine whether there were other cells that retain viral genomes but do not express viral RNAs. However, since 90% of the infected cells are GFP^+^ at 1 dpi, these cells would constitute a very small minority of the infected cells.

Our findings have two important implications. First, the observation that viral gene expression is first turned on, and then turned off, suggests that there is a mechanism for shutting off viral genomes that are actively engaged in transcription. This may be due to expression of viral genes that facilitate establishment of latency (37, 41, 46–49, 66, 72, 73). However, these genes are also expressed in models of lytic infection and could not act directly to repress viral gene expression, since they are not localized to the nucleus. An attractive hypothesis that would account for the highly restricted tropism for HCMV latency is that there is a host defense response that shuts off viral transcription specifically in myeloid lineage cells.

Second, shedding of HCMV is often observed in healthy, immunocompetent individuals, suggesting that the virus reactivates with high frequency (74). The finding that HCMV establishes latency in cells following activation of transcription raises the possibility that these genomes may retain a memory of prior activation and may therefore be poised to reactivate readily under the appropriate conditions. Interestingly, previous studies have shown that HCMV chromatin is specifically marked by H3 lysine 4 methylation following replication of viral DNA in fibroblasts (75). It is tempting to speculate that there may be cell-type-specific differences in histone modifications bound to postreplicative viral DNA that affect the potential to establish latency and to reactivate.

### Viral transcription in latency.

Previous studies showed that some viral genes that are expressed during lytic infection, particularly UL138 and US28, facilitate establishment of latency in myeloid progenitor cells (37, 41, 46–49, 66, 72, 73). However, analysis of expression of these genes in latently infected cells has been contradictory and confusing. Some studies have shown that some genes are selectively expressed in latency (12, 14, 37, 46, 47, 51–53). In contrast, studies using unbiased transcriptome analyses to detect viral transcripts in both experimental and natural latency have identified many genes expressed under conditions of latency, and none of these show specific expression of some RNAs in the absence of all other lytic genes (11, 39, 42, 43, 76). This raises the possibility that detection of these transcripts is due to the presence of a small population of lytically infected cells. With the exception of the noncoding miR-UL148D microRNA (37), most previous studies have not included analyses of the RNA/DNA ratio to demonstrate differences in transcriptional activity between lytic genes and latency-associated lytic genes. With the exception of LUNA (14), there has been no analysis of histones bound to latency-associated genes in latently infected cells to support the idea that they are selectively active. We have investigated expression of US28 and UL138 as well as the noncoding RNA2.7 in the Kasumi-3 model. We find that the pattern of expression of these genes parallels that of other lytic genes, including UL122, UL123, UL54, and UL32, and analysis of the RNA/DNA ratio shows that transcription of all of these genes is repressed in latency. We suggest that this type of analysis should be included in future studies to demonstrate latency-specific expression of a gene. Collectively, our studies and others are consistent with the idea that these genes facilitate establishment of latency through changes in the relative abundance of viral transcripts but that they are shut off when latency is established. This situation would be analogous to that of EBV latency, where the EBNA genes are expressed in naive B cells but are shut off in memory B cells, which are the long-term site of latency (77), and HSV, where ICP0 is initially expressed to turn on LAT expression and facilitate heterochromatinization of viral genomes (67).

### What is the role of myeloid cell differentiation in reactivation of HCMV?

Previous studies using primary CD34-derived or monocyte-derived dendritic cells (MoDCs) showed that LPS-induced reactivation of latent HCMV required differentiation to a dendritic cell phenotype (9, 13, 20, 30, 32, 52, 78). Reactivation in immature dendritic cells in response to LPS occurred through induction of IL-6 and activation of extracellular signal-regulated kinases (ERKs) and mitogen- and stress-activated kinases (MSKs) (52, 79). However, the factors that were used to differentiate dendritic cells in these models are also mediators of inflammation, and it is therefore difficult to distinguish the roles of a general inflammatory response, which could occur in many cell types, from a dendritic cell-specific mechanism of reactivation. In contrast, Kasumi-3 cells, like many transformed cell lines, are refractory to the normal cues that induce differentiation and are therefore more suitable for studies seeking to dissect the roles of these different processes. Our studies show that differentiation is not required for reactivation in response to TNF-α.

Previous studies showed that, in latently infected primary CD34^+^ hematopoietic progenitor cells, viral genomes are bound to KAP-1 and that KAP-1 is required to establish latency through heterochromatinization and transcriptional silencing (40). Reactivation could be induced by treatment of the cells with chloroquine, an agent that induces phosphorylation of KAP-1 through activation of ATM, or by knockdown of KAP-1, and this effect was potentiated by cotreatment with TNF-α. These cells retained expression of CD34, suggesting that differentiation was not required for reactivation in this model, although expression of dendritic cell markers was not analyzed (40).

ATM is the master regulator of the response to double-stranded DNA breaks (DSBs) (80). ATM is recruited to damage sites by the Mre11-Rad50-Nbs1 (MRN) complex, where it phosphorylates a variety of substrates that facilitate DNA repair, including KAP-1. KAP-1 is a multifunctional protein whose activity is regulated through posttranslational modifications (81). The SUMOylated form of KAP-1 acts as a repressor of transcription through recruitment of factors that mediate H3K9 deacetylation/methylation. ATM-mediated phosphorylation of KAP-1 leads to changes in its interaction partners that result in decondensation of chromatin to allow repair of damaged sites, and this is especially important in repair of breaks that occur in regions of heterochromatin (81–83). The results from Rauwel et al. (40) therefore suggest that reactivation of HCMV can be induced through chromatin remodeling induced by activation of a DNA damage response independently of differentiation to dendritic cells. Cotreatment with TNF-α potentiated reactivation in response to chloroquine, suggesting that inflammatory mediators that activate NF-κB can act synergistically with chromatin remodeling to induce reactivation of HCMV. Our studies showed that TNF-α was sufficient to induce reactivation in Kasumi-3 cells without inducing differentiation and that this correlated with activation of NF-κB, as well as ATM and KAP-1. Further studies will be required to fully understand the roles of differentiation, inflammation, and chromatin remodeling in reactivation of HCMV. However, the results of our studies, as well as those of others, suggest that the mechanisms of reactivation of HCMV are not necessarily limited to a dendritic cell-specific pathway but may be induced by more general pathways of inflammation and cellular damage.

## MATERIALS AND METHODS

### Cells and reagents.

Kasumi-3 and MRC-5 cells were obtained from the ATCC. Human recombinant TNF-α was obtained from PeproTech (catalog no. 300-01A). Fluorescently conjugated monoclonal antibodies used in this study are listed in Table 1. TB40/E*wt*-GFP (48) viral stocks were produced and their titers were determined as described in Text S1 in the supplemental material.

10.1128/mBio.01560-18.1 TEXT S1  Supplemental methods. Download TEXT S1, PDF file, 0.1 MB. Copyright © 2018 Forte et al. 2018 Forte et al. This content is distributed under the terms of the Creative Commons Attribution 4.0 International license.

**TABLE 1:** Antibodies used in this study

Marker	Clone	Supplier (catalog no.)
CD34	581	BD Biosciences
CD45	HI30	BD Biosciences
CD1c	F10/21A3	BD Biosciences
CD11c	B-ly6	BD Biosciences
CD14	MOP9	BD Biosciences
CD15	HI98	BD Biosciences
CD64	10.1	BD Biosciences
IE1/2	CH160	Virusys
NF-κB p65	L8F6	Cell Signaling Technology (9460)
Phospho-NF-κB p65 (Ser536)	93H1	Cell Signaling Technology
ATM	D2E2	Cell Signaling Technology (2873)
Phospho-ATM (S1981)	D25E5	Cell Signaling Technology (13050)
KAP-1	EPR5216	Abcam (109287)
Phospho-KAP-1 (S824)	EPR5248	Abcam (133440)
γ-H2AX (S139)	EP854(2)Y	Abcam (195189)

### Infection of Kasumi-3 cells.

Kasumi-3 cells were infected with HCMV strain TB40/E*wt*-GFP (48) (MOI of 1 to 2) using centrifugal enhancement of infection, as described in Text S1 in the supplemental material (84). GFP^+^ and GFP^−^ cells were sorted on a FACSAria-6 laser cell sorter (BD Biosciences). Infected cells were cultured for 13 days to establish latency, with medium changes every 2 to 3 days. For reactivation studies, cells were treated with 5 ng/ml TNF-α at 14 dpi for 3 days at 37°C. A 50% tissue culture infective dose (TCID_50_) assay was used to determine the infectious titer of infected cell supernatants.

### RNA and DNA extraction.

DNA was isolated with the Arcturus Pico pure DNA extraction kit (Thermo Fisher Scientific) as recommended by the manufacturer. RNA was purified with the Direct-zol RNA miniprep kit (Zymo Research) with a DNase digestion step according to the manufacturer’s instructions.

### Real-time PCR analysis.

For all analyses, mock-infected cells were analyzed in parallel with infected cells. These samples were uniformly positive for cellular genes but negative for viral genes. TaqMan assays specific for viral genes were custom designed by Life Technologies (Table 2). Relative viral DNA and RNA quantity was analyzed by the threshold cycle (2^−ΔΔ*CT*^) method, using predesigned assays (Life Technologies) for RNase P (catalog number 4403326) or glyceraldehyde-3-phosphate dehydrogenase (GAPDH), respectively, as the normalization controls. This method was validated by determining that the efficiencies of the target and reference genes were approximately equal (85) as described in the supplemental methods (Text S1 and Fig. S5 and S6). Previous studies have shown that GAPDH is an appropriate normalization control for RNA analysis of CMV-infected cells (86), and we verified that GAPDH *C*_*T*_ values are stable following infection of Kasumi-3 cells (Fig. S7). TaqMan gene expression assays purchased from Life Technologies were used for analysis of cellular RNAs (Hs00174128_m1 for TNF-α and Hs99999905_m1 for GAPDH).

10.1128/mBio.01560-18.6 FIG S5  Analysis of the efficiencies of amplification of viral genes versus RNase P. Viral genes and the cellular gene RNase P were amplified in samples prepared from serial dilutions of DNA isolated from lytically infected MRC-5 fibroblasts. The Δ*C*_*T*_ values (*C*_*T*_ of viral gene − *C*_*T*_ of RNase P) for each dilution were calculated and plotted against the log nanograms of DNA. Download FIG S5, PDF file, 0.1 MB. Copyright © 2018 Forte et al. 2018 Forte et al. This content is distributed under the terms of the Creative Commons Attribution 4.0 International license.

10.1128/mBio.01560-18.7 FIG S6  Analysis of the efficiencies of amplification of viral RNAs versus GAPDH. Viral RNAs and cellular GAPDH RNA were amplified in samples prepared from serial dilutions of cDNA prepared from RNA isolated from lytically infected MRC-5 fibroblasts. The Δ*C*_*T*_ values (*C*_*T*_ of viral gene − *C*_*T*_ of GAPDH) for each dilution were calculated and plotted against the log nanograms of cDNA. Download FIG S6, PDF file, 0.1 MB. Copyright © 2018 Forte et al. 2018 Forte et al. This content is distributed under the terms of the Creative Commons Attribution 4.0 International license.

10.1128/mBio.01560-18.8 FIG S7  Validation of GAPDH as a normalization control in HCMV-infected Kasumi-3 cells. Data show average *C*_*T*_ values ± standard deviation for GAPDH at various times after infection. *n =* 4. Download FIG S7, PDF file, 0.1 MB. Copyright © 2018 Forte et al. 2018 Forte et al. This content is distributed under the terms of the Creative Commons Attribution 4.0 International license.

**TABLE 2:** TaqMan assays for analysis of viral DNA and RNA

Assay name	Sequence (5′ to 3′)		
	Forward primer	Reverse primer	Reporter
UL122	GGCTCACCTCGTCAATCTTGA	CAACGAGAAGGTACGCAATATCATG	CCCCTTCTGCACACCC
UL123	GCACCCGACAAAACTCACTTAAGA	TGACGCTTGTATGATGACCATGT	ACGGGTCCATCTCTC
UL54	GAAACATAGCCGCCACAGAAC	ACGAATAGTGTTGCCGTGTCA	AACGCCGCTATCATCT
UL32	GGTGAAACGCGGATCTTGAC	GCTCACGGAGACCAGAGG	CTGTCGGAATCCTCG
UL138	CTTCCTCCCAACGGACCAA	CGGACAGACGATACCGTTTCT	ACGGCCCGATGAGATC
US28	CCGATCATCCTCAACGTAGAACT	CGGTAGTAGCAGTAGCTGATGAC	CATGCTCGGTGCTTTC
2.7-kb RNA	TCACCGTTTTCTCTCTTCTCTCTCT	TCGAAGAATGAAAGACGACGATGATT	CCGCTCCTGCCACCCG

### Immunophenotyping.

Cells were stained with antibodies specific for CD34, CD64, CD14, CD15, CD11c, CD1c, and IE1/2 (CH160) (Table 1) as described in Text S1 in the supplemental material. Data on live cells were acquired on a 6-laser BD LSRFortessa SORP flow cytometer and analyzed using FlowJo software (version 9).

### Cytokine stimulation and intracellular phosphoprotein analysis.

Kasumi-3 cells were treated or not treated with 5 ng/ml TNF for 30 min at 37°C, fixed, permeabilized, and washed according to previously described protocols (87, 88). Cells were stained with antibodies specific for p65 (NF-κB), p-p65 (pNF-κB), ATM, p-ATM, KAP-1, p-KAP-1, or γ-H2AX (Table 1). Antibodies were validated using published protocols (89) as described in Text S1 and Fig. S8. Data were acquired on a BD LSRFortessa SORP flow cytometer and analyzed using FlowJo (version 9). The fraction of responding cells for each population was determined as described in Text S1 as previously described (88, 89).

10.1128/mBio.01560-18.9 FIG S8  Antibody staining validation. (A) Representative flow cytometric analysis of HeLa cells, untreated (red) or treated with human TNF-α (20 ng/ml) and calyculin A (100 nM) for 15 min (blue), using phospho-NF-κB p65 (Ser536) rabbit monoclonal antibody and total NF-κB p65. (B and C) Representative flow cytometric analysis of HCT116 treated with 200 nM newborn calf serum (NCS), using phospho-ATM (S1981), phospho-KAP-1 (S824) monoclonal antibody, ATM, and total KAP-1 monoclonal antibody (blue) compared to untreated control cells (red). Download FIG S8, PDF file, 0.9 MB. Copyright © 2018 Forte et al. 2018 Forte et al. This content is distributed under the terms of the Creative Commons Attribution 4.0 International license.
