# Supplementary figures and images for: Tumor Necrosis Factor Alpha Induces Reactivation of Human Cytomegalovirus Independently of Myeloid Cell Differentiation following Posttranscriptional Establishment of Latency
Source: mBio. 2018 Sep 11;9(5):e01560-18. doi: 10.1128/mBio.01560-18 (PMC6134100; doi:10.1128/mBio.01560-18)

A

UL123 DNA

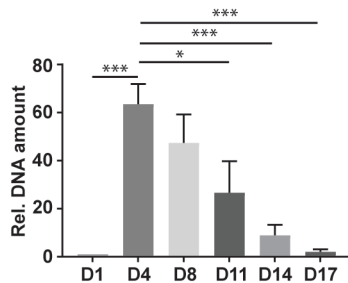

UL54 DNA

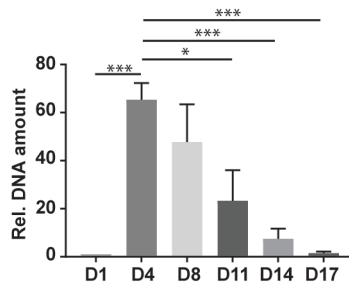

UL32 DNA

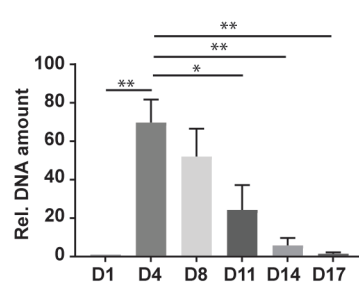

B

UL123 RNA

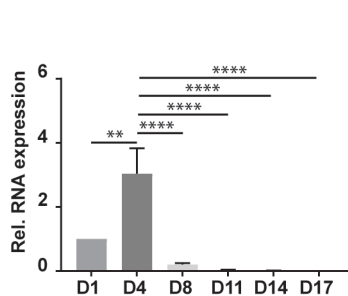

UL54 RNA

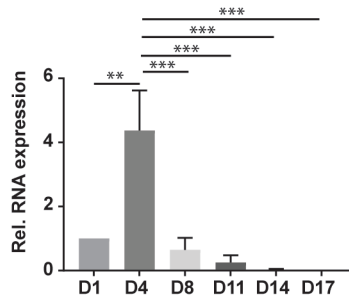

UL32 RNA

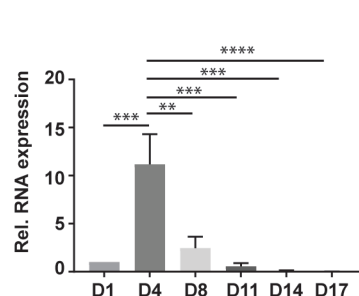

Fig. S1

FIG. 9

Supplement: FIG S1 [file mbo004184056sf1.pdf]

A

## UL122 RNA

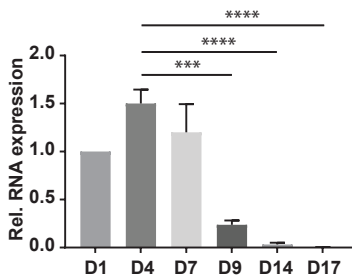

## UL122 DNA

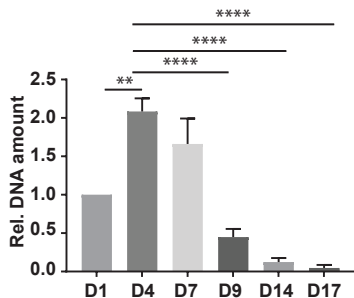

B

## UL54 RNA

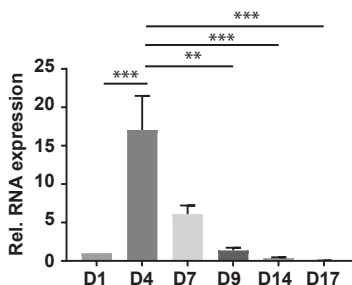

## UL54 DNA

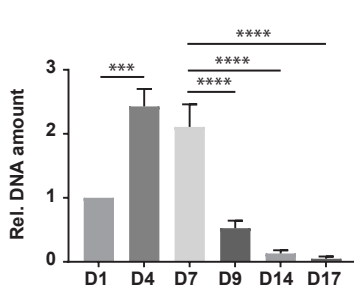

C

## UL32 RNA

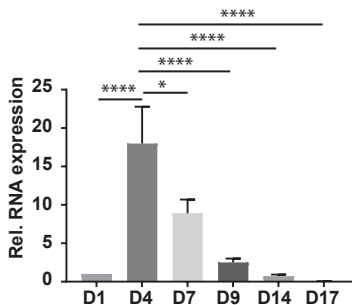

## UL32 DNA

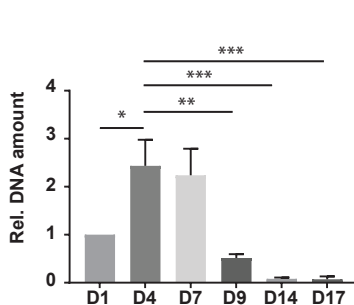

Fig. S2

Supplement: FIG S2 [file mbo004184056sf2.pdf]

**A**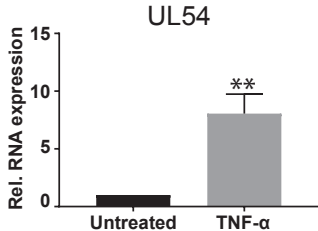**B**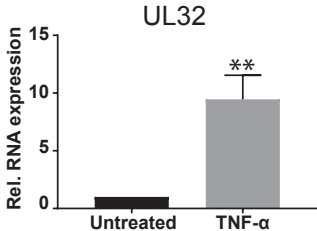**Fig. S3**

Supplement: FIG S3 [file mbo004184056sf3.pdf]

A

Uninfected

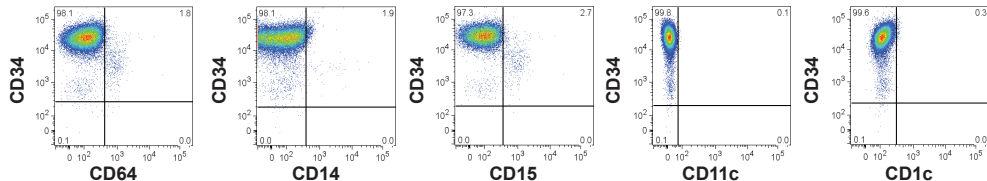

B

Infected D17 Untreated

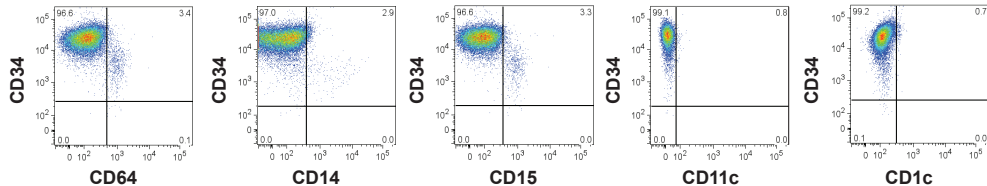

Fig. S4

Supplement: FIG S4 [file mbo004184056sf4.pdf]

**UL122 vs RNaseP**

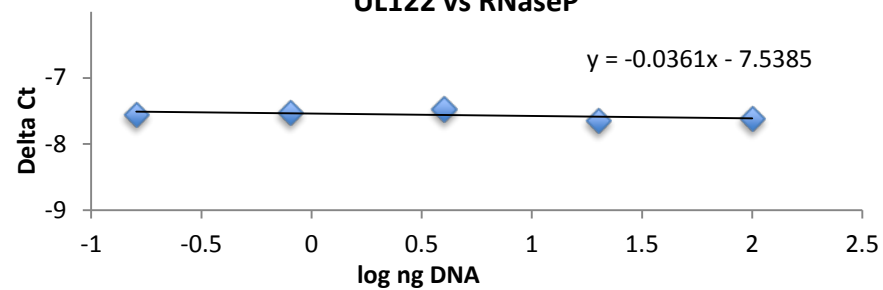

**UL123 vs RNaseP**

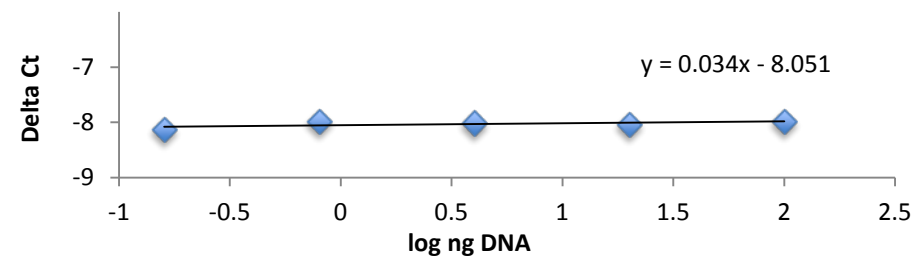

**UL54 vs RNaseP**

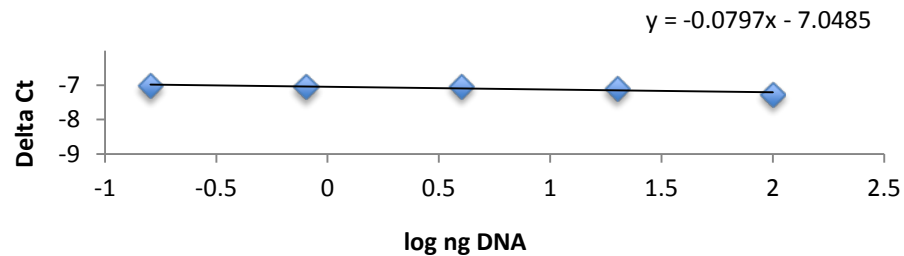

**UL32 vs RNaseP**

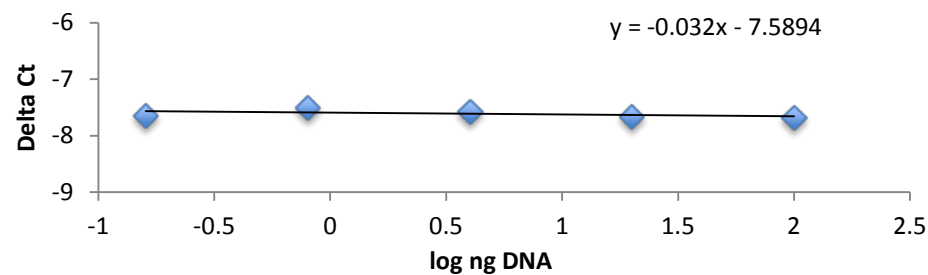

**US28 vs RNaseP**

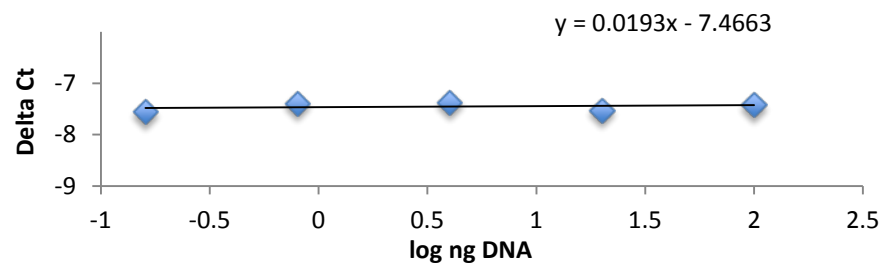

**UL138 vs RNaseP**

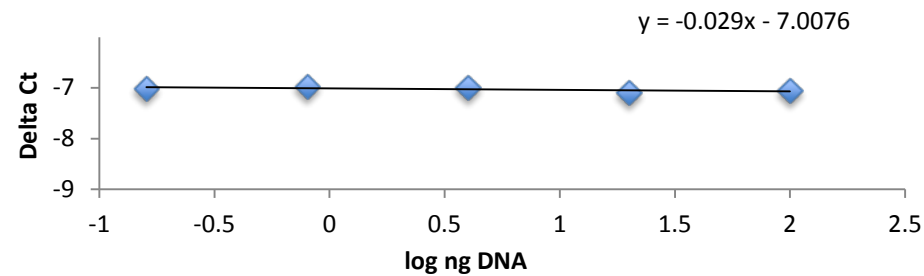

**RNA 2.7**

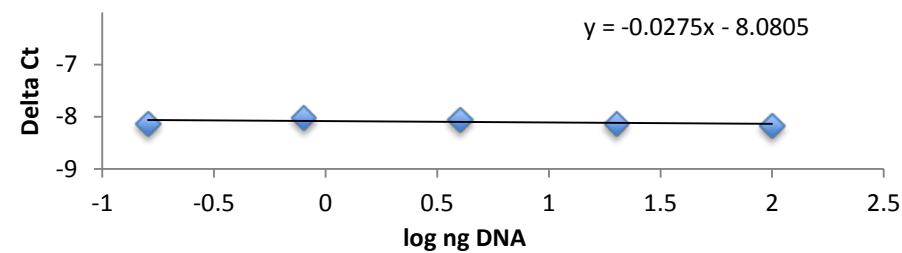

**Fig. S5**

Supplement: FIG S5 [file mbo004184056sf5.pdf]

**UL122 vs GAPDH**

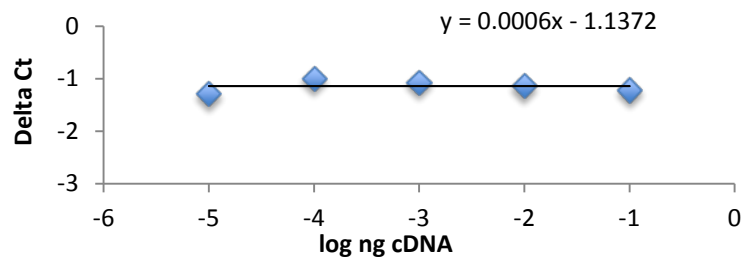

**UL123 vs GAPDH**

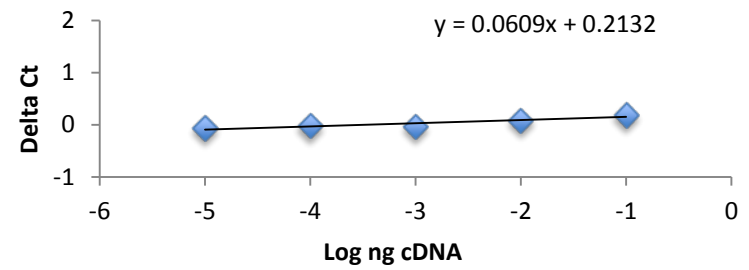

**UL32 vs GAPDH**

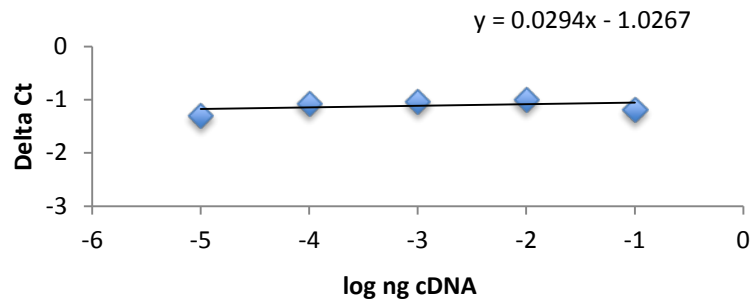

**UL54 vs GAPDH**

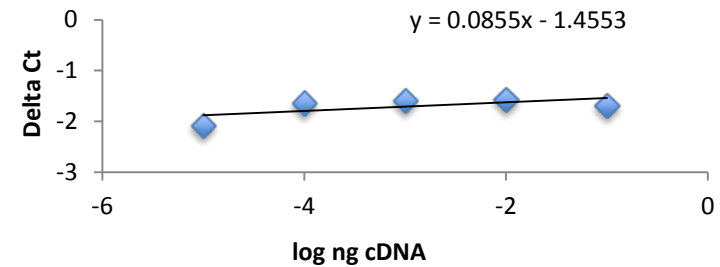

**RNA 2.7 vs GAPDH**

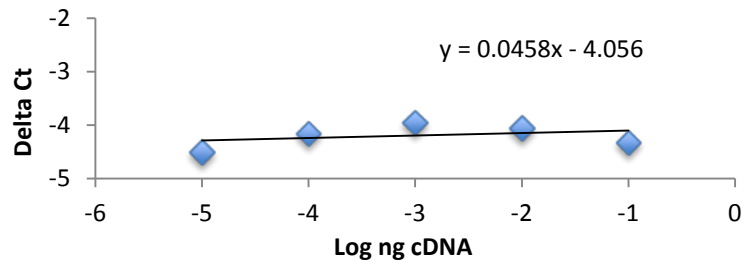

**UL138 vs GAPDH**

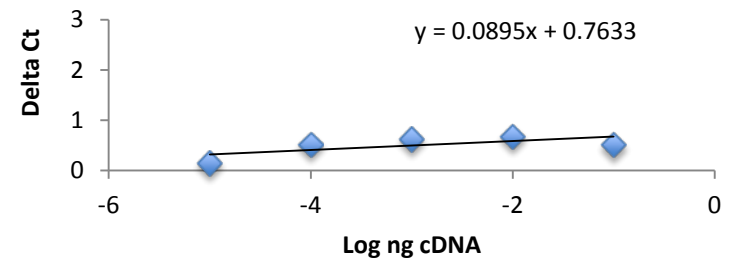

**US28 vs GAPDH**

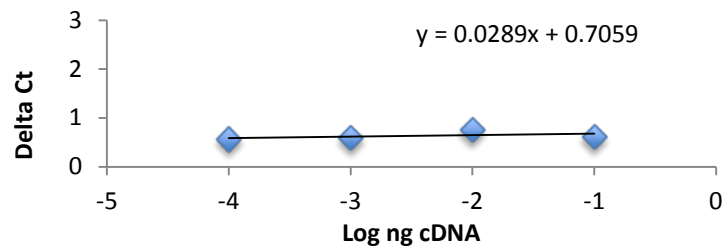

**Fig. S6**

Supplement: FIG S6 [file mbo004184056sf6.pdf]

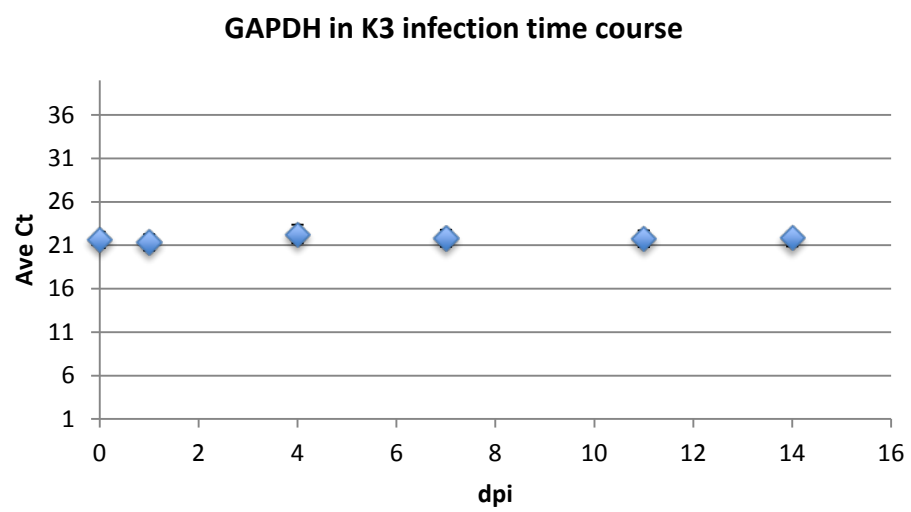

Fig. S7

Supplement: FIG S7 [file mbo004184056sf7.pdf]

A

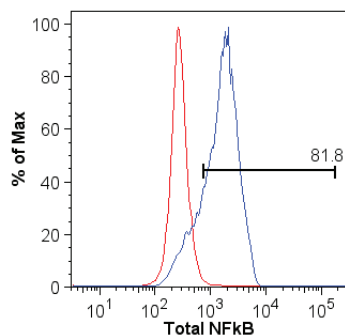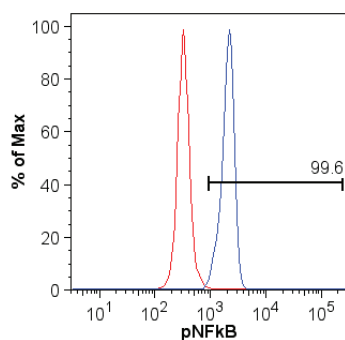

B

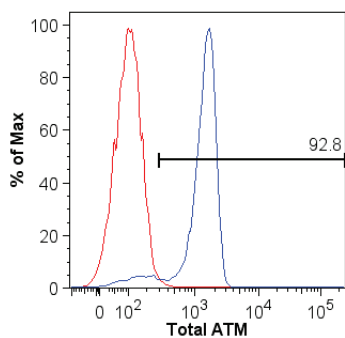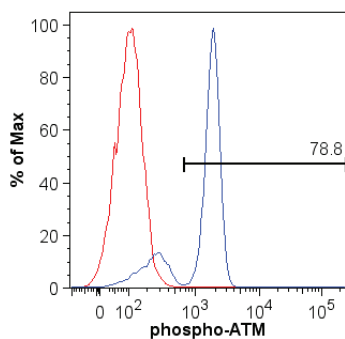

C

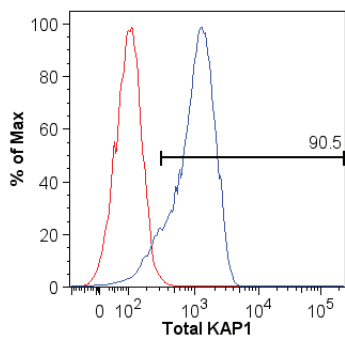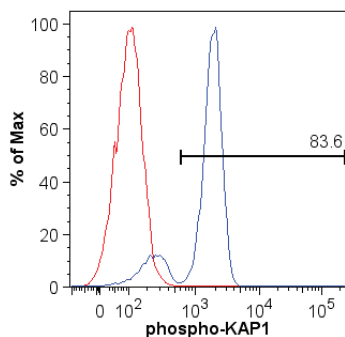

Fig. S8

Supplement: FIG S8 [file mbo004184056sf8.pdf]
