# Supplementary material for: Tumor Necrosis Factor Alpha Induces Reactivation of Human Cytomegalovirus Independently of Myeloid Cell Differentiation following Posttranscriptional Establishment of Latency
Source: mBio. 2018 Sep 11;9(5):e01560-18. doi: 10.1128/mBio.01560-18 (PMC6134100; doi:10.1128/mBio.01560-18)
Supplement: TEXT S1 [file mbo004184056s1.pdf]

Supplementary Methods.

Virus stocks. TB40/E*wt*-GFP (1) viral stocks were produced by electroporation of BAC DNA into MRC-5 fibroblasts with pGCN-pp71 expression vector. Culture media was collected, cleared of cellular debris by centrifugation and concentrated by ultracentrifugation on a sorbitol cushion (2). Viral titers were determined using the 50% tissue culture infective dose ($TCID_{50}$) limiting dilution assay after two weeks in culture. Titers were calculated from duplicate 96-well plates by scoring the GFP+ wells using the method of Reed and Muench (3).

Infection of Kasumi-3 cells: Kasumi-3 cells were infected with HCMV strain TB40/E*wt*-GFP at high concentration ($2x10^7$ cells/ml) using an MOI of 1-2 and centrifugal enhancement of infection (1000x*g* for 30 minutes at 25°C), then diluted to $1.25x10^6$ cells/ml with fresh media and incubated at 37°C for additional 23 hours. 1 day post-infection (dpi), extracellular virus was inactivated by treatment with Citric Acid solution (40mM sodium citrate, 10 mM potassium chloride, 135 mM sodium chloride, pH 3.05) as previously described (4).

Real time PCR analysis. TaqMan assays specific for viral genes were custom-designed by Life Technologies (Table 2). Relative viral DNA quantity was analyzed by the 2-(ΔΔCt) method, using a pre-designed assay for RNaseP as the normalization control (Life Technologies, catalog number 4403326). This method was validated by determining that the efficiencies of the target and reference genes were approximately equal, as previously described (5). Briefly, viral genes and the cellular gene RNaseP were amplified in samples prepared from serial dilutions of DNA isolated from lytically infected MRC-5 fibroblasts. The ΔCt values ($C_T$viral gene – C_TRNaseP) for each dilution were calculated and plotted against the log ng DNA. The slope of this plot was <0.1 (Fig. S5). Relative viral RNA expression was analyzed by the 2(-ΔΔCt) method using GAPDH as the normalization control. We validated that the efficiency of amplification of

GAPDH was similar to that of each viral gene, using serial dilutions of cDNA from lytically infected MRC-5 cells as the template (Fig. S6). Previous studies have shown that GAPDH is an appropriate normalization control for RNA analysis of CMV-infected cells (6), and we verified that GAPDH Ct values are stable following infection of Kasumi-3 cells (Fig. S7). TaqMan gene expression assays purchased from Life Technologies were used for analysis of cellular RNAs (Hs00174128_m1 for TNF-α and Hs99999905_m1 for GAPDH).

Immunophenotyping. For cell surface immunophenotyping, cells were washed twice in FACS buffer (2% BSA in phosphate-buffered saline (PBS)), stained with the appropriate antibodies for 30 min at 4°C, washed twice and resuspended in PBS for analysis by flow cytometry. For phenotyping of reactivated cells (including intracellular targets) and signaling analyses, cells were washed, fixed at a final concentration of 4% formaldehyde and permeabilized with 80% methanol per previously defined protocols (7, 8). Cells were then washed twice with FACS buffer, re-suspended in 75 µl blocking serum solution and stained for 1 hour on ice with CD34, CD64, CD14, CD15, CD11c, CD1c and IE 1/2 (CH160) for phenotyping. Data on live cells was acquired on 6-laser BD LSRFortessa SORP flow cytometer, equipped with 355nm, 405nm, 488nm, 553nm, 635nm and 690nm lasers with standard filter sets, and analyzed using FlowJo software (version 9).

Validation of flow cytometric analysis of antibodies. Phospho-ATM, total ATM, phospho-KAP1, and total KAP-1 antibody staining was validated by analyzing neocarzinostatin (NCS)-induced phosphorylation in HCT 116 cells. Cells were either untreated or treated with 200nM NCS for 30 minutes, at 37°C followed by immediate fixation with 4% methanol free formaldehyde, followed by 80% methanol permeabilization. Following fix-permeabilization the cells were washed and stained with a pre-titered volume of antibody (Fig. S8). Phospho-p65

(NFkB) antibody staining was validated by TNF-a-induced NFkB phosphorylation of HeLa cells. HeLa cells were either untreated or treated with 100 nM Calyculin A, an inhibitor of protein phosphatase 1 (PP1) and protein phosphatase 2A (PP2A-C) for 10 minutes followed by 20ng/ml of human TNF-a for 5 minutes at 37°C, followed by immediate fixation and permeabilization. Following these, the cells were washed, and processed with a pre-titered volume of phospho-NF-kB p65 (Ser563) rabbit mAb (Fig. S8).

Cytokine stimulation and intracellular phospho-protein analysis: Aliquots of $3x10^6$ uninfected or HCMV-infected Kasumi-3 cells were pre-incubated at 37^{0}C for 30', then treated or untreated with 5 ng/ml TNF for 30' at 37°C. Samples were then fixed, permeabilized and washed according to previously described protocols (7, 8). For intracellular staining, cells were washed twice with PBS and, re-suspended in 75 μl blocking serum solution and stained with antibodies specific for CD34, p65 (NF-κB), p-p65 (pNF-κB), ATM, p-ATM, KAP-1, p-KAP-1 or γ-H2AX (Table 1). Suspensions were incubated for 1 hour on ice, washed twice with ice cold PBS/BSA, centrifuged (600Xg) 6' at 4^{0}C, and re-suspended in 300 μl ice cold PBS for data acquisition. Data were acquired on BD LSRFortessa SORP flow cytometer and analyzed using FlowJo (version 9). The fraction of responding cells for each population was determined from single parameter phospho-protein histograms based on a fixed region set on the inhibitor/untreated control at ~3%. The magnitude of response was calculated as previously described (8, 9), by comparing the stimulated tube to inhibitor control tube and ratioing MFI of the responding cells within each discrete phenotypic population using the following formula:

$$Treated\left(\frac{MFI\ Treated\ Stained}{MFI\ Treated\ Unstained}\right) / \ Untreated\left(\frac{MFI\ Untreated\ Stained}{MFI\ Untrearted\ Unstained}\right)$$

Supplementary references

1. **Umashankar M, Petrucelli A, Cicchini L, Caposio P, Kreklywich CN, Rak M, Bughio F, Goldman DC, Hamlin KL, Nelson JA, Fleming WH, Streblow DN, Goodrum F.** 2011. A novel human cytomegalovirus locus modulates cell type-specific outcomes of infection. PLoS Pathog **7:**e1002444.
2. **Stinski MF.** 1976. Human cytomegalovirus: glycoproteins associated with virions and dense bodies. J Virol **19:**594-609.
3. **Reed LJ, Muench H.** 1938. A simple method of estimating fifty per cent endpoints. Am J Hyg **27:**493-497.
4. **Umashankar M, Goodrum F.** 2014. Hematopoietic long-term culture (hLTC) for human cytomegalovirus latency and reactivation. Methods Mol Biol **1119:**99-112.
5. **Livak KJ, Schmittgen TD.** 2001. Analysis of relative gene expression data using real-time quantitative PCR and the 2(-Delta Delta C(T)) Method. Methods (Duluth) **25:**402-408.
6. **Watson S, Mercier S, Bye C, Wilkinson J, Cunningham AL, Harman AN.** 2007. Determination of suitable housekeeping genes for normalisation of quantitative real time PCR analysis of cells infected with human immunodeficiency virus and herpes viruses. Virol J **4:**130.
7. **Chow S, Minden MD, Hedley DW.** 2006. Constitutive phosphorylation of the S6 ribosomal protein via mTOR and ERK signaling in the peripheral blasts of acute leukemia patients. Exp Hematol **34:**1183-1191.
8. **Marvin J, Swaminathan S, Kraker G, Chadburn A, Jacobberger J, Goolsby C.** 2011. Normal bone marrow signal-transduction profiles: a requisite for enhanced detection of signaling dysregulations in AML. Blood **117:**e120-130.
9. **Gao J, Swaminathan S, Pai N, Johnson Z, Chen YH, Peterson L, Goolsby C.** 2015. Flow cytometric detection of altered signaling in myelodysplastic syndrome and cytopenia. Leuk Res **39:**1396-1404.
